# *CCNB1* and *AURKA* are critical genes for prostate cancer progression and castration-resistant prostate cancer resistant to vinblastine

**DOI:** 10.3389/fendo.2022.1106175

**Published:** 2022-12-19

**Authors:** Xi Chen, Junjie Ma, Xin’an Wang, Tong Zi, Duocheng Qian, Chao Li, Chengdang Xu

**Affiliations:** ^1^ Department of Urology, Tongji Hospital, School of Medicine, Tongji University, Shanghai, China; ^2^ Department of Urology, The Second Affiliated Hospital of Jiaxing University, Jiaxing, Zhejiang, China; ^3^ Department of Urology, Shanghai Fourth People’s Hospital, Affiliated to Tongji University School of Medicine, Shanghai, China

**Keywords:** vinblastine resistant, CCNB1, AURKA, castration-resistant prostate cancer, cancer development, bioinformatic analysis

## Abstract

**Background:**

Prostate cancer (PCa) is a common malignancy occurring in men. As both an endocrine and gonadal organ, prostate is closely correlated with androgen. So, androgen deprivation therapy (ADT) is effective for treating PCa. However, patients will develop castration-resistant prostate cancer (CRPC) stage after ADT. Many other treatments for CRPC exist, including chemotherapy. Vinblastine, a chemotherapeutic drug, is used to treat CRPC. However, patients will develop resistance to vinblastine. Genetic alterations have been speculated to play a critical role in CRPC resistance to vinblastine; however, its mechanism remains unclear.

**Methods:**

Various databases, such as Gene Expression Omnibus (GEO), The Cancer Genome Atlas (TCGA) and Chinese Prostate Cancer Genome and Epigenome Atlas (CPGEA), were used to collect the RNA-sequence data of PCa and CRPC patients and vinblastine-resistant PCa cells. Using online tools, Metascape and TIMER, the pathways and immune infiltration associated with vinblastine resistance-related genes in PCa were analyzed. The function of these genes was verified in clinical samples and CRPC cells.

**Results:**

Using GSE81277 dataset, we collected the RNA-sequence data of vinblastine sensitive and resistant LNCaP cells and found nine genes (*CDC20*, *LRRFIP1*, *CCNB1*, *GPSM2*, *AURKA*, *EBLN2*, *CCDC150*, *CENPA* and *TROAP*) that correlated with vinblastine resistance. Furthermore, *CCNB1*, *GPSM2* and *AURKA* were differently expressed between normal prostate and PCa tissues, even influencing PCa progression. The GSE35988 dataset revealed that *CCNB1* and *AURKA* were upregulated in PCa and CRPC samples. Various genes were also found to affect the survival status of PCa patients based on TCGA. These genes were also related to immune cell infiltration. Finally, we verified the function of *CCNB1* and *AURKA* and observed that they were upregulated in PCa and CRPC clinical samples and increased the sensitivity of CRPC cells to vinblastine.

**Conclusion:**

*CCNB1* and *AURKA* are central to CRPC resistance to vinblastine and affect PCa progression.

## 1 Introduction

Prostate cancer (PCa) is a common disease occurring in older men and has the highest incidence and second-highest mortality rate in the USA (1). In China, the morbidity and mortality rates of PCa have been increasing rapidly (2). To date, many effective methods have been used to treat PCa, including surgical intervention, androgen deprivation therapy (ADT) and chemotherapy (3). As a hormone-sensitive endocrine and gonadal organ, the progression of PCa is closely related with androgen. This is also the reason that ADT is the first-line therapy method for treating PCa; however, patients undergoing ADT progress to the castration-resistant PCa (CRPC) stage (4). Moreover, chemotherapy, a second-line treatment, can be used in treating both hormone-sensitive PCa (HSPC) and CRPC (5). However, patients with chemotherapy also develop resistance to the treatment. The mechanism of resistance to chemotherapy in patients with CRPC remains unclear.

Vinblastine is a type of chemotherapeutic drug that can regulate spindle microtubule formation and inhibit nuclear division at metaphase. Additionally, it can also inhibit the viability of the RNA synthesis enzyme, thereby killing the cells in the G1 phase (6). Vinblastine has been widely used in the treatment of many solid cancers, such as lung, breast and ovarian cancers. Vinblastine is also used in HSPC and CRPC treatments (7–9). However, vinblastine treatment eventually, in most cases, leads to vinblastine resistance in patients. Genetic alteration is hypothesised to be one of the main reasons for the development of vinblastine resistance in patients with PCa and CRPC; however, its underlying mechanism remains unknown.

Hence, this study aims to identify the specific gene alterations in patients with CRPC who show resistance to vinblastine. In this study, we used a gene dataset, GSE81277, to identify the differentially expressed genes between normal and vinblastine-resistant PCa LNCaP cells. Furthermore, the role of these genes in affecting the PCa development was analyzed and immune cell infiltration in PCa were also analyzed. We found two key genes, *CCNB1* and *AURKA* were upregulated in PCa and CRPC samples and influenced the sensitivity of CRPC cells to vinblastine. So, we thought that *CCNB1* and *AURKA* may play an important role in CRPC resistant to vinblastine.

## 2 Materials and methods

### 2.1 Data sourcing

We collected three gene datasets, GSE81277, GSE21034 and GSE35988, from the Gene Expression Omnibus (GEO) database (http://www.ncbi.nlm.nih.gov/geo/). GSE81277 included the RNA sequence data of LNCaP PCa cells resistant to vinblastine. Three vinblastine sensitive and three resistant samples were obtained from GSE81277. Furthermore, GSE21034 and GSE35988 included the RNA and clinical data of patients with PCa. Additionally, the clinical data of patients with PCa were collected from both The Cancer Genome Atlas (TCGA) (http://cancergenome.nih.gov/) and Chinese Prostate Cancer Genome and Epigenome Atlas (CPGEA) (http://www.cpgea.com) databases.

### 2.2 Data handing

The primary RNA-sequence data obtained from different databases were normalized using R software (version 4.0.3). Based on the document comments, the expression matrix including probe ID was substituted by the corresponding gene ID. The genes with |log2FC > 1| and *P* < 0.05 were considered as critical genes. The genes were reflected in volcano map made by R software “Enhancedvolcano” package. Additionally, the clinical data from different databases were downloaded for further study.

### 2.3 Pathways analysis and protein-protein interaction network

The pathways of the enriched hub genes were analyzed using the online tool Metascape (http://metascape.org/), and the bubble map was constructed using R software “ggplot2” package. Furthermore, the PPI network was constructed using STRING (https://cn.string-db.org/).

### 2.4 Online tool

Online tools, such as UALCAN (http://ualcan.path.uab.edu/), gene expression profiling interactive analysis (GEPIA) (http://www.gepia.cancer-pku.cn/) and Tumour Immune Estimation Resource (TIMER) (https://cistrome.shinyapps.io/timer/) were used for analysis.

### 2.5 Tumour stage and survival analysis

Based on the clinical data from different databases, the expression of hub genes in different tumour stages was analyzed. Additionally, using GEPIA the hub genes that influence overall survival (OS) and disease-free survival (DFS) in patients with PCa were also analyzed.

### 2.6 Immune immersion analysis

The correlation and mutation type of the identified hub genes and immune cells in PCa were analyzed using TIMER.

### 2.7 Clinical specimen collection

Clinical PCa and CRPC specimens were collected from Tongji Hospital, School of Medicine, Tongji University. The collection method was approved by the Ethics Committee of Tongji Hospital, School of Medicine, Tongji University (SBKT-2021-220). Patients who provided the samples were informed of the experiment and gave informed consent.

### 2.8 Cell culture and drug treatment

PCa cell lines were purchased from the Chinese Academy of Science Cell Bank (Shanghai, China). The human CRPC cell lines C4-2 and 22Rv1 were cultured in Roswell Park Memorial Institute (RPMI) 1640 medium (Catalog No. R8758, Sigma, Darmstadt, Germany) containing 10% fetal bovine serum (FBS) (Catalog No. 10091, Gibco, Thermo Fisher Scientific, Waltham, MA, USA). The cells were cultured in a humid environment with 5% CO2 and 95% air at 37°C. Vinblastine (Catalog No. S4505) was purchased from SelleckChem (Houston, TX, USA). The CRPC cells were treated with vinblastine (3.25 nmol/L) for 24 h.

### 2.9 Cell transfection and lentivirus production

Cell transfection assays were performed with Lipofectamine 2000 (Catalog No. 11668019, Thermo Fisher Scientific). shRNA lentivirus was constructed for specific gene knockdowns. The shRNAs were purchased from the Youze Biotechnology Company. Additionally, blank control lentivirus (shControl) without knockdown specific genes was also constructed by the Youze Biotechnology Company. The shRNA sequence was as follows: shCCNB1: GCAGCACCTGGCTAAGAATGCAGCACCTGGCTAAGAAT and shAURKA: CCGGCCTGTCTTACTGTCATTCGAACTCGAGTTCGAATGACAGTAAGCAGGTTTTTG.

### 2.10 RNA extraction and qRT-PCR

The total RNA was extracted from CRPC cell lines utilizing TRIzol Reagent (Sigma–Aldrich, St. Louis, MO, USA, Catalog No. T9424). cDNA was transcribed using the reverse transcription kit (Advantage^®^ RT-for-PCR Kit, Takara Bio Inc., Kusatsu, Japan, Catalog No. 639505). Finally, we measured the volume of cDNA using a real-time PCR kit (TB Green^®^ Premix Ex Taq™ II, Takara Bio Inc., Catalog No. RR420A) according to the manufacturer’s instructions. The primers of CCNB1, AURKA and GAPDH are shown in **Table 1**. The 2^−ΔΔCt^ method was used to quantify mRNA expression levels.

**Table 1:** Primers used for the qRT-PCR.

Gene Name	Primer sequence
CCNB1	Forward: 5′-GCACTTTCCTCCTTCTCA-3′
	Reverse: 5′-CGATGTGGCATACTTGTT-3
AURKA	Forward: 5′-ACAGGTCTGGCTGGCCGTTGGC-3′
	Reverse: 5′-GGCGCACACCGCGCGCAGGCG-3′
GAPDH	Forward: 5-GGAGCGAGATCCCTCCAAAAT-3′
	Reverse: 5′-GGCTGTTGTCATACTTCTCATGG-3′

### 2.11 Antibodies

Rabbit monoclonal anti-CCNB1 antibody (Catalog No. ab156447), anti-AURKA antibody (Catalog No. ab108353) and anti-GAPDH antibody (Catalog No. ab9485) were purchased from Abcam (Abcam UK, Cambridge, UK).

### 2.12 Western blot

Tissue samples and cell line proteins were extracted with RIPA lysis buffer. Protein samples were treated with Dual Colour Protein Loading Buffer (Thermo Fisher Scientific, Waltham, MA, USA). Sodium dodecyl-sulfate polyacrylamide gel electrophoresis (10%) was used to separate proteins, which were then transferred to nitrocellulose membranes (Merck KGaA, Darmstadt, Germany). Protein-Free Rapid Blocking Buffer (Thermo Fisher Scientific) was utilized to block the membranes. Then, the membranes were incubated at 4°C overnight with primary antibodies against CCNB1 (1:1000), AURKA (1:1000) and GAPDH (1:1000) (Abcam UK, Cambridge, UK). On the second day, the membranes were washed thrice using 1×TBST (10 min/cycle). Then, the membranes were incubated at normal temperature for 1.5 h with a matched secondary antibody (Catalog No. A0208, HRP-labeled Goat Anti-Human IgG (H+L), Beyotime Biotechnology, Shanghai, China). Finally, the membranes were exposed to X-ray film.

### 2.13 Cell proliferation assay

Cell proliferation ability was detected using Cell counting kit-8 (CCK-8) (Dojindo, Japan). Briefly, cells were placed in 96-well plates (3000 cells/well) and cultured with 200 µL RPMI 1640 + 10% FBS for 0 h, 24 h, 48 h or 72 h. After culturing, cells were detected using CCK-8, following the manufacturer’s instructions. Absorbance at 450 nm was measured using a spectrophotometer (LD942, Beijing, China).

### 2.14 Statistical analysis

The matrix data were analyzed using R version 4.0.3 (Institute for Statistics and Mathematics, Vienna, Austria; https://www.r-project.org). Comparisons between two groups were performed using the Wilcoxon test, and the Kruskal–Wallis test was employed for comparisons between more than two groups. Hazard ratios (HRs), 95% confidence interval (95% CI) and P values were used as statistical metrics. Twotailed,P < 0.05 was deemed as statistically significant.

## 3 Results

### 3.1 Nine hub genes correlated with vinblastine resistance in LNCaP PCa cells

**Figure 1:**
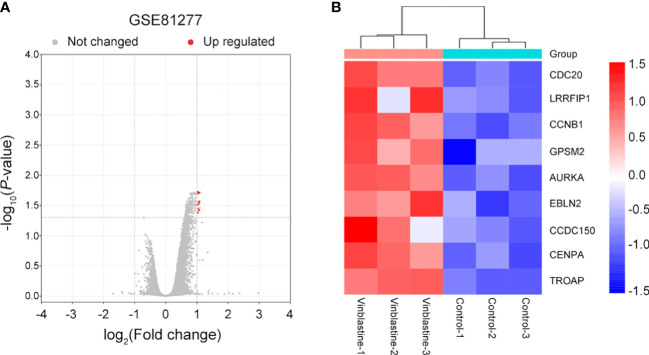
Nine vinblastine resistance-related genes were found from GSE81277. **(A)** The volcano map reflects the differently expressed genes in GSE81277. **(B)** The heatmap reflected the expression of nine hub genes in different datasets of GSE81277.

The potential hub genes that correlated with PCa resistance to vinblastine were identified. In the GEO database, we found a gene dataset, GSE81277, which included the RNA-sequence data of both vinblastine-sensitive and -resistant LNCaP cells. Subsequently, the volcano map revealed nine hub genes (*CDC20*, *LRRFIP1*, *CCNB1*, *GPSM2*, *AURKA*, *EBLN2*, *CCDC150*, *CENPA* and *TROAP)* from LNCaP PCa cell samples that correlated with vinblastine resistance (**Figure 1A**). Further, we constructed a heat map to reflect the specific expression of each hub gene in the GSE81277 dataset (**Figure 1B**).

### 3.2 The pathways and PPI network of the enriched nine hub genes

**Figure 2:**
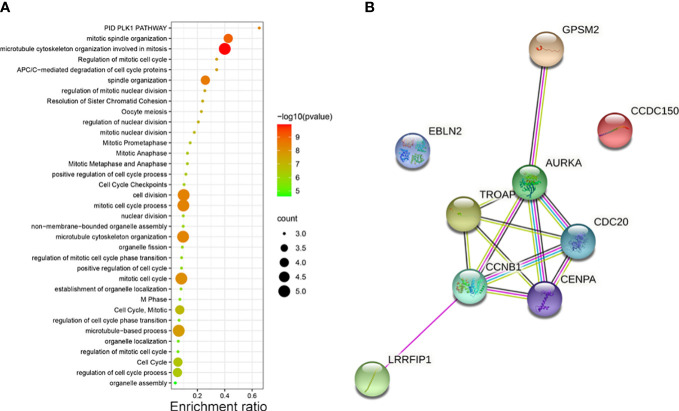
**(A)** The pathways of vinblastine resistance-related genes depending on Metascape. **(B)** The PPI network reflects the association between each hub genes.

The potential pathways and the correlation of each hub gene were analyzed using Metascape. The pathways enriched by the hub genes are illustrated in a bubble map. We found these genes mainly enriched in “microtubule cytoskeleton organization involved in mitosis” (**Figure 2A**). Then, using STRING, we constructed the PPI network to find the correlation between each hub gene. The network revealed that apart from *EBLN2* and *CCDC150*, each hub gene correlated with other genes (**Figure 2B**).

### 3.3 Vinblastine resistance-related hub gene expressions in PCa samples

**Figure 3:**
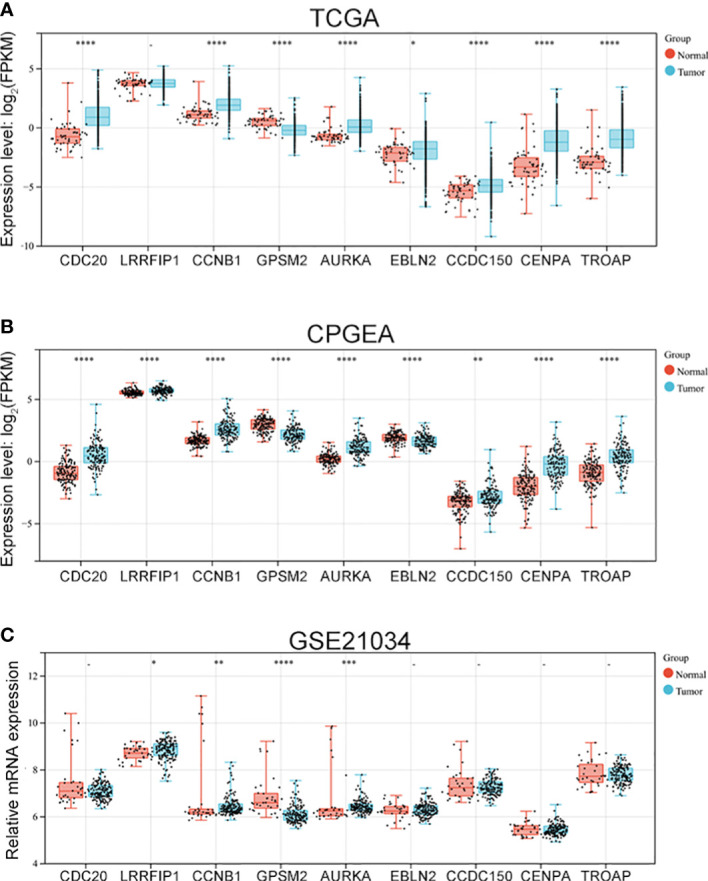
The expression of vinblastine resistance-related genes in PCa patients from different databases. Expression of nine vinblastine resistance-related hub genes in PCa patients from TCGA **(A)** database, CPGEA **(B)** database, and GSE21034 **(C)** dataset. – represents no statistical differences. * represents *P*<0.05, ** represents *P*<0.01, *** represents *P*<0.001, **** represents *P*<0.0001.

After identifying the nine hub genes that were associated with vinblastine resistance in PCa, we examined the expression of these genes in PCa. We collected the sequence data of patients with PCa from different databases, such as TCGA, CPGEA and GEO. In TCGA, apart from *LRRFIP1*, all other gene expressions changed in PCa. Moreover, only *GPSM2* was downregulated whereas other genes were upregulated in PCa (**Figure 3A**). As the TCGA data included samples from Western populations, we further tested the expression of these hub genes in different populations, including the Asian population. Using the CPGEA database, we collected the RNA-sequence data of the Chinese population. The data revealed a change in the expressions of these nine genes in Chinese patients with PCa. Furthermore, *GPSM2* and *EBLN2* were downregulated whereas other hub genes were upregulated (**Figure 3B**). Finally, the GSE21034 dataset was used to verify the results. In GSE21034, we found that the expression of *LRRFIP1*, *CCNB1*, *GPSM2* and, *AURKA* changed in PCa samples, with *GPSM2* being downregulated (**Figure 3C**). As methylation level can affect the function of genes, we further examined the methylation level of these nine genes in the TCGA database. However, data on *EBLN2* could not be found and thus, only the methylation level of eight genes was tested. The methylation level of *GPSM2*, *EBLN2*, *CCDC150* and *TROAP* showed a statistically significant change compared to the other genes (**Figure S1**).

### 3.4 Vinblastine resistance-related hub genes affect PCa progression

**Figure 4:**
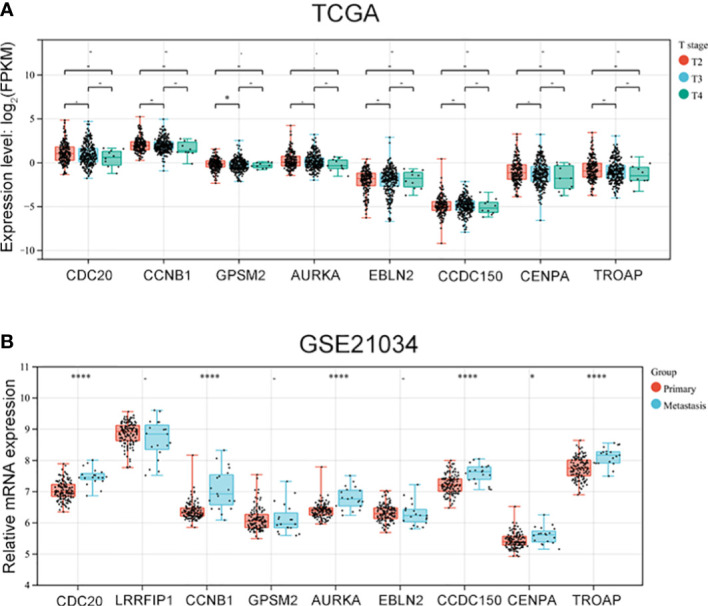
The expression of vinblastine resistance-related genes in different tumor stage from different databases. **(A)** The expression of eight vinblastine resistance-related genes in different T stage from TCGA database. **(B)** The expression of nine vinblastine resistance-related hub genes in primary and metastasis PCa patients from GSE21034 datasets. – represents no statistical differences. * represents *P*<0.05, **** represents *P*<0.0001.

As vinblastine resistance-related hub genes have been reported to influence PCa occurrence, we further evaluated these genes’ role in PCa progression. As the Tumour-Node-Metastasis (TNM) staging has been widely used to determine the severity of PCa (10), we evaluated the expression of these nine hub genes in different TNM tumour stages. Depending on the clinical data from different databases, the function of these vinblastine resistance-related hub genes in PCa progression was analyzed. In the TCGA database, we found that *GPSM2* expression was downregulated when PCa progressed from the T2 stage to the T3 stage (**Figure 4A**). As *LIRRFIP1* was not differentially expressed between normal and tumour tissues, *LIRRFIP1* was not included in the study. However, the other genes did not significantly affect the primary tumour (T) stage (**Figure 4A**). In Chinese patients with PCa, the nine genes also did not show a significant effect on the T stage (**Figure S2A**). Furthermore, the nine genes also did not significantly influence node metastasis (**Figures S2B, C**). Then, using the clinical data from GSE21034, we classified the patients with PCa into a primary tumour group and metastasis group based on the occurrence of distant metastasis. *CDC20*, *CCNB1*, *AURKA*, *CDCC150* and *TROAP* were observed to influence distant metastasis (**Figure 4B**).

### 3.5 Verification of the expression of vinblastine resistance-related genes in CRPC samples

**Figure 5:**
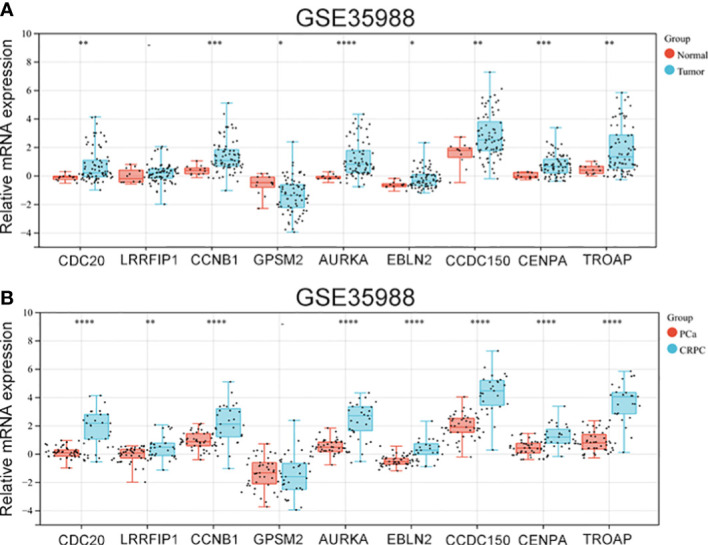
The expression of vinblastine resistance-related genes in GSE35988 datasets. **(A)** The expression of nine vinblastine resistance-related hub genes between normal and tumor samples from GSE35988. **(B)** The expression of nine vinblastine resistance-related hub genes between PCa and CRPC samples from GSE35988. – represents no statistical differences. * represents *P*<0.05, ** represents *P*<0.01, *** represents *P*<0.001, **** represents *P*<0.0001.

The GSE35988 dataset was used to verify the above results. In GSE35988, apart from *LRRFIP1*, all other genes influenced PCa occurrence (**Figure 5A**). Further, using the GSE35988 dataset, we classified patients with PCa into a primary tumour group and a CRPC group. Apart from *GPSM2*, other genes were upregulated in the CRPC samples (**Figure 5B**).

### 3.6 Vinblastine resistance-related hub genes affect survival status in patients with PCa

**Figure 6:**
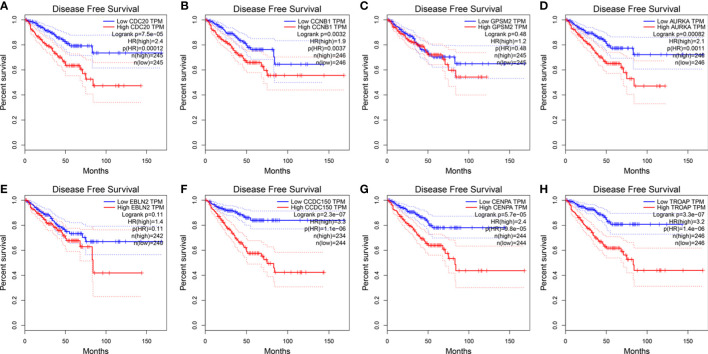
The expression of vinblastine resistance-related genes with PCa patients DFS status from TCGA database. **(A)** *CDC20* **(B)** *CCNB1* **(C)** *GPSM2* **(D)** *AURKA* **(E)** *EBLN2* **(F)** *CCDC150* **(G)** *CENPA* **(H)** *TROAP*.

The influence of the eight hub genes on the survival status of patients with PCa was evaluated using GEPIA. *LRRFIP1* did not significantly affect PCa occurrence; therefore, it was excluded from the study process. Apart from *GPSM2* and *EBLN2*, all other genes were observed to influence the DFS of patients with PCa (**Figure 6**). Furthermore, *CDC20* and *CDCC150* influenced the OS of patients with PCa (**Figure S3**).

### 3.7 Association of vinblastine resistance-related hub genes with immune infiltration in PCa

Immune infiltration has been reported to play an important role in PCa (11). Hence, the correlation between these vinblastine resistance-related hub genes and immune cells in PCa was analyzed using TIMER. However, *EBLN2* could not be identified using TIMER, thereby excluding it from the study. Apart from *LRRFIP1* and *TROAP*, all other gene mutation types were associated with immune cell infiltrations in PCa (**Figure S4**).

**Figure 7:**
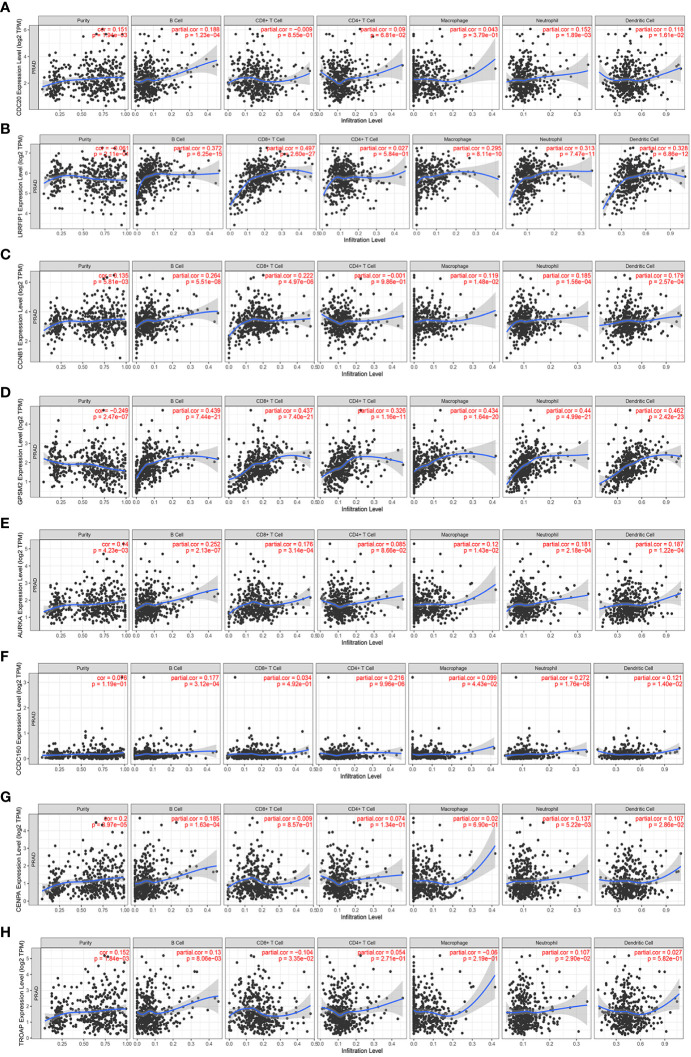
The correlation between the expression of vinblastine resistance-related genes and immune cell infiltration from TIMER webtool. **(A)** *CDC20* **(B)** *LRRFIP1* **(C)** *CCNB1* **(D)** *GPSM2* **(E)** *AURKA* **(F)** *CCDC150* **(G)** *CENPA* **(H)** *TROAP*.

Next, the specific correlation between these gene expressions and immune cells in PCa was evaluated. *LRRFIP1* was not observed to be associated with CD4+T cells in PCa (**Figure 7B**). Moreover, *AURKA* was not correlated with Purity cell, and CD8+T cell in PCa (**Figure 7E**). Furthermore, *CCDC150* was not correlated with CD4+T cell, CD8+T cell and Macrophage cell in PCa (**Figure 7F**). Additionally, *TROAP* was not correlated with CD4+T cells and Macrophage cells in PCa (**Figure 7G**). However, other genes showed a correlation with all immune cells in PCa (**Figure 7**).

### 3.8 Validation of the expression and function of vinblastine resistance-related hub genes in CRPC samples and cells

Finally, the function of these hub genes was verified using clinical samples and PCa cells. Depending on that the above results, *CCNB1* and *AURKA* were upregulated in vinblastine-resistant PCa cells, PCa samples and CRPC samples. Additionally, they also affected the patient’s survival status, and even PCa progression. hence, they were selected as the study objectives and thought they may critical for CRPC resistant to vinblastine. C4-2 PCa cells, which were cultured from LNCaP cells and grown in an environment without androgen, were considered as a cell line model of CRPC and another PCa cell line: 22Rv1 which can also growth without androgen were used in the study (12). Further, these two types of cells are sensitive to vinblastine.

**Figure 8:**
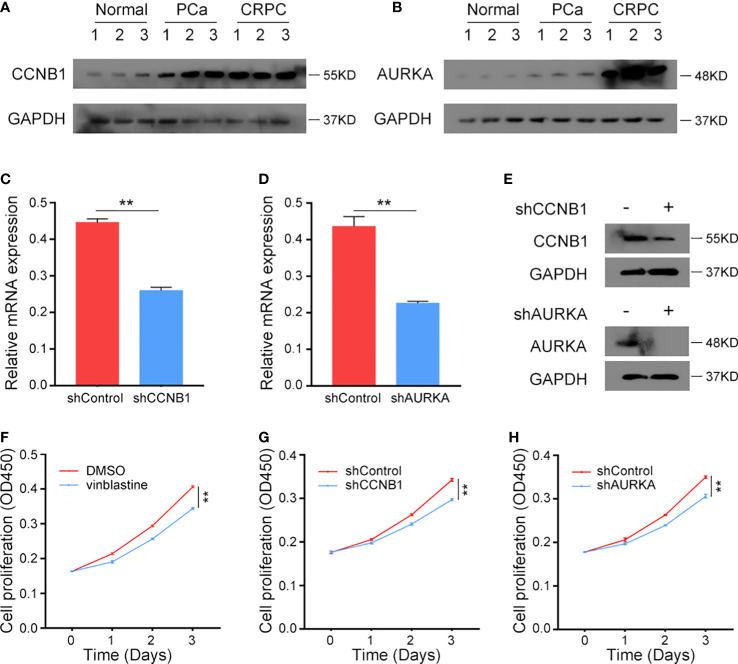
The expression *CCNB1* and *AURKA* in clinical samples and the function of *CCNB1* and *AURKA* to C4-2 cells in vinblastine. **(A, B)** The protein level of CCNB1 **(A)** and AURKA **(B)** in normal, primary PCa, and CRPC samples. **(C, D)** The mRNA level of CCNB1 **(C)** and AURKA **(D)** when different lentivirus transfected into C4-2 CRPC cells. **(E)** The protein level of CCNB1 and AURKA when different lentivirus transfected into C4-2 CRPC cells. **(F)** The cell proliferation level of C4-2 cells when C4-2 cells treated by DMSO or vinblastine. **(G, H)** The cell proliferation level of C4-2 cells after different lentivirus transfected into C4-2 CRPC cells with vinblastine treatment. **(G)** shCCNB1 **(H)** shAURKA. ** represents *P*<0.01.

The protein levels of CCNB1 and AURKA were analyzed in clinical normal prostate tissues, primary PCa samples and CRPC samples. These genes were found to be upregulated in tumour tissues and highly expressed in CRPC samples (**Figures 8A, B**), indicating their role in the occurrence of both PCa and CRPC. Next, we constructed CCNB1 and AURKA knockdown CRPC cell lines. The knockdown lentivirus decreased the expression of CCNB1 and AURKA at both mRNA and protein levels in C4-2 cells (**Figures 8C–E**) and can also inhibit the expression of CCNB1 and AURKA protein in 22Rv1 cells (**Figure S5A**). Then, the C4-2 and 22Rv1 cell lines were treated with vinblastine for 24 h to determine their proliferation ability. We observed that vinblastine decreased cell proliferation of C4-2 (**Figure 8F**). Further same results were observed in 22Rv1 cells (**Figure S5B**). Next, we transfected the specific knockdown lentivirus into C4-2 and 22Rv1 cells to determine the influence of the two genes on CRPC cells proliferation ability with vinblastine treatment. CCNB1 and AURKA knockdown decreased the proliferation ability of the C4-2 cells (**Figures 8G, H**) and 22Rv1 cells (**Figure S5C, D**). Thus, CCNB1 and AURKA have a potential role in the occurrence of PCa and CRPC and can influence CRPC resistant to vinblastine.

## 4 Discussion

With the increase in life span, the incidence of PCa among men is increasing rapidly (2). The progression of PCa is correlated with androgen closely and this is the reason that ADT can treat PCa effectively. As the first-line therapy method, ADT can effectively prolong the life span of patients with PCa (13). However, ADT treatment eventually leads to the progression of primary PCa tumours to the CRPC stage (14). Moreover, chemotherapy can also be used in the treatment of PCa and CRPC (6). Some chemotherapy drugs like docetaxel and vinblastine have been reported to be useful in treating PCa, achieving good curative effects (5). Hence, elucidating the mechanism of chemotherapeutic resistance to chemotherapy drugs is of clinical importance in CRPC treatment.

In 1999, a Phase II clinical trial reported that vinblastine improved the symptoms in patients with advanced PCa and had a good tolerance (15). Furthermore, vinblastine was also reported to improve the effect of Estramustine phosphate in treating PCa (16). Additionally, vinblastine has also been used to treat CRPC (5, 7, 17). It improves the effects of the various drugs used in CRPC treatment. Studies have reported that vinblastine improves the function of docetaxel and prednisone in treating CRPC (8, 18, 19). Moreover, vinblastine treatment of PCa and CRPC has also been reported *in vitro*. It decreases the proliferation and induces the apoptosis of PCa cell lines, such as LNCaP and DU145 (20, 21). Thus, vinblastine effectively treats PCa and CRPC; however, resistance to vinblastine eventually occurs.

Genetic alterations could play a vital role in CRPC resistance to vinblastine. Hence, in this study, we aimed to identify the potential genes that correlated with CRPC resistance to vinblastine. Nine hub genes were found to be upregulated in vinblastine-resistant LNCaP cells. Moreover, on examining their expression and clinical value, two hub genes, *CCNB1* and *AURKA*, were upregulated in vinblastine-resistant CRPC cells, PCa clinical samples and CRPC samples. This indicates that these two hub genes are not only important to vinblastine resistance but also in CRPC. Finally, functional analyses of *CCNB1* and *AURKA* revealed that these two genes affected the sensitivity of CRPC cells to vinblastine, indicating their role in the occurrence of PCa and resistance to drugs. Therefore, these two genes’ alterations may be the reason for CRPC resistant to vinblastine.

Cyclin B1 (*CCNB1*), which is essential for cell cycle progression through mitosis, is overexpressed in various cancers compared with normal cells and tissues like breast cancer and non-small cell lung cancer (22–24). The overexpression of *CCNB1* has also been indicated as a poor outcome in some patients with cancer (24, 25). In patients with PCa, high *CCNB1* expression often occurred with a high tumour grade (26). Additionally, some studies also reported that patients with a high *CCNB1* expression are likely to experience tumour metastasis and have a poor prognosis (27, 28). Consistent with previous results, this study also observed that *CCNB1* affected PCa progression and prognosis. Similarly, another study reported that the high expression of *CCNB1* could be a potential reason for PCa resistance to docetaxel (29). Similar to *CCNB1*, Aurora kinase A (*AURKA*) is also a cell cycle-regulated kinase that is involved in microtubule formation or stabilization at the spindle pole during chromosome segregation (30). Genetic analysis reveals that *AURKA* is upregulated in PCa tissues, especially in neuroendocrine PCa tissues (31). In PCa, *AURKA* is considered an oncogene. Its oncogenic function has been correlated with N-myc (32, 33). Furthermore, a Phase II study reported that an *AURKA* inhibitor, Alisertib, could treat PCa (34). Moreover, Alisertib has also been reported to enhance the effect of docetaxel in PCa treatment (35). Additionally, *AURKA* is considered a critical factor for solid tumour resistance to chemotherapy drugs (36). In this study, we also proved that these two genes were important in CRPC and even CRPC resistant to vinblastine.

However, this study has many limitations. First, the nine hub genes identified are from LNCaP PCa cells. Although LNCaP is a PCa cell line cultured from metastasis node tissues obtained from patients with PCa (37), it is less representative than clinical samples. Therefore, results observed in PCa cells may differ from those in clinical samples. Second, although the expression and function of the two hub genes could be important in the occurrence of both PCa and CRPC clinical samples and PCa cell lines, the study size was small hence, the results cannot be generalised. Therefore, further experiments with large samples are required to validate our findings. Third, although vinblastine can be used to treat CRPC, it is not commonly used in clinical. So, the clinical value of this study is limited. However, this study still contributes to defining CRPC resistant to vincristine. To the best of our knowledge, this study is the first to identify the potential genetic alterations that occur in CRPC resistance to vinblastine.

## 5 Conclusion

Nine hub genes (*CDC20*, *LRRFIP1*, *CCNB1*, *GPSM2*, *AURKA*, *EBLN2*, *CCDC150*, *CENPA* and *TROAP)* that may play a vital role in PCa resistance to vinblastine were identified and they also corelated with PCa progression. Furthermore, two hub genes, *CCNB1* and *AURKA* are important factors for CRPC resistant to vinblastine.

## Data availability statement

The original contributions presented in the study are included in the article/supplementary material. Further inquiries can be directed to the corresponding authors.

## Ethics statement

The studies involving human participants were reviewed and approved by Ethic committee of Tongji Hospital, School of Medicine, Tongji University. The patients/participants provided their written informed consent to participate in this study.

## Author contributions

XC and JM put forward the idea of the article, wrote the manuscript and analyzed the data. TZ collected the data from public database. XW finished the RT-qPCR, Western blot and CCK-8 experiments. CL help collecting the clinical specimens. DQ and CX revised the manuscript. All authors contributed to the article and approved the submitted version.

## References

[1] Siegel RL Miller KD Fuchs HE Jemal A . Cancer statistics, 2021. CA Cancer J Clin (2021) 71(1):7–33. doi: 10.3322/caac.21654 33433946

[2] Chen X Ma J Xu C Wang L Yao Y Wang X . Identification of hub genes predicting the development of prostate cancer from benign prostate hyperplasia and analyzing their clinical value in prostate cancer by bioinformatic analysis. Discovery Oncol (2022) 13(1):54. doi: 10.1007/s12672-022-00508-y PMC9243208 35768705

[3] Chen X Wu Y Wang X Xu C Wang L Jian J . CDK6 is upregulated and may be a potential therapeutic target in enzalutamide-resistant castration-resistant prostate cancer. Eur J Med Res (2022) 27(1):105. doi: 10.1186/s40001-022-00730-y 35780240 PMC9250190

[4] Heidenreich A Bastian PJ Bellmunt J Bolla M Joniau S van der Kwast T . EAU guidelines on prostate cancer. part II: Treatment of advanced, relapsing, and castration-resistant prostate cancer. Eur Urol (2014) 65(2):467–79. doi: 10.1016/j.eururo.2013.11.002 24321502

[5] Arsov C Winter C Rabenalt R Albers P . Current second-line treatment options for patients with castration resistant prostate cancer (CRPC) resistant to docetaxel. Urol Oncol (2012) 30(6):762–71. doi: 10.1016/j.urolonc.2010.02.001 20884252

[6] Nader R El Amm J Aragon-Ching JB . Role of chemotherapy in prostate cancer. Asian J Androl (2018) 20(3):221–9. doi: 10.4103/aja.aja_40_17 PMC5952475 29063869

[7] Oudard S Caty A Humblet Y Beauduin M Suc E Piccart M . Phase II study of vinorelbine in patients with androgen-independent prostate cancer. Ann Oncol (2001) 12(6):847–52. doi: 10.1023/A:1011141611560 11484963

[8] Koletsky AJ Guerra ML Kronish L . Phase II study of vinorelbine and low-dose docetaxel in chemotherapy-naive patients with hormone-refractory prostate cancer. Cancer J (2003) 9(4):286–92. doi: 10.1097/00130404-200307000-00011 12967139

[9] Sweeney CJ Monaco FJ Jung SH Wasielewski MJ Picus J Ansari RH . A phase II Hoosier oncology group study of vinorelbine and estramustine phosphate in hormone-refractory prostate cancer. Ann Oncol (2002) 13(3):435–40. doi: 10.1093/annonc/mdf029 11996476

[10] Paner GP Stadler WM Hansel DE Montironi R Lin DW Amin MB . Updates in the eighth edition of the tumor-Node-Metastasis staging classification for urologic cancers. Eur Urol (2018) 73(4):560–9. doi: 10.1016/j.eururo.2017.12.018 29325693

[11] Strasner A Karin M . Immune infiltration and prostate cancer. Front Oncol (2015) 5:128. doi: 10.3389/fonc.2015.00128 26217583 PMC4495337

[12] Zegarra-Moro OL Schmidt LJ Huang H Tindall DJ . Disruption of androgen receptor function inhibits proliferation of androgen-refractory prostate cancer cells. Cancer Res (2002) 62(4):1008–13. 11861374

[13] Culig Z Santer FR . Androgen receptor signaling in prostate cancer. Cancer Metastasis Rev (2014) 33(2-3):413–27. doi: 10.1007/s10555-013-9474-0 24384911

[14] Ross RW Xie W Regan MM Pomerantz M Nakabayashi M Daskivich TJ . Efficacy of androgen deprivation therapy (ADT) in patients with advanced prostate cancer: association between Gleason score, prostate-specific antigen level, and prior ADT exposure with duration of ADT effect. Cancer (2008) 112(6):1247–53. doi: 10.1002/cncr.23304 18293426

[15] Fields-Jones S Koletsky A Wilding G O'Rourke M O'Rourke T Eckardt J . Improvements in clinical benefit with vinorelbine in the treatment of hormone-refractory prostate cancer: a phase II trial. Ann Oncol (1999) 10(11):1307–10. doi: 10.1023/A:1008315106697 10631457

[16] Hudes G Einhorn L Ross E Balsham A Loehrer P Ramsey H . Vinblastine versus vinblastine plus oral estramustine phosphate for patients with hormone-refractory prostate cancer: A Hoosier oncology group and fox chase network phase III trial. J Clin Oncol (1999) 17(10):3160–6. doi: 10.1200/JCO.1999.17.10.3160 10506613

[17] Morant R Hsu Schmitz SF Bernhard J Thurlimann B Borner M Wernli M . Vinorelbine in androgen-independent metastatic prostatic carcinoma–a phase II study. Eur J Cancer (2002) 38(12):1626–32. doi: 10.1016/S0959-8049(02)00145-4 12142053

[18] Robles C Furst AJ Sriratana P Lai S Chua L Donnelly E . Phase II study of vinorelbine with low dose prednisone in the treatment of hormone-refractory metastatic prostate cancer. Oncol Rep (2003) 10(4):885–9. doi: 10.3892/or.10.4.885 12792740

[19] Tralongo P Bollina R Aiello R Di Mari A Moruzzi G Beretta G . Vinorelbine and prednisone in older cancer patients with hormone-refractory metastatic prostate cancer. a phase II study. Tumori (2003) 89(1):26–30. doi: 10.1177030089160308900106 12729357 10.1177/030089160308900106

[20] Hossein G Zavareh VA Fard PS . Combined treatment of androgen-independent prostate cancer cell line DU145 with chemotherapeutic agents and lithium chloride: Effect on growth arrest and/or apoptosis. Avicenna J Med Biotechnol (2012) 4(2):75–87. 23408470 PMC3558206

[21] Levrier C Rockstroh A Gabrielli B Kavallaris M Lehman M Davis RA . Discovery of thalicthuberine as a novel antimitotic agent from nature that disrupts microtubule dynamics and induces apoptosis in prostate cancer cells. Cell Cycle (2018) 17(5):652–68. doi: 10.1080/15384101.2017.1356512 PMC5976206 28749250

[22] Pines J . Mitosis: a matter of getting rid of the right protein at the right time. Trends Cell Biol (2006) 16(1):55–63. doi: 10.1016/j.tcb.2005.11.006 16337124

[23] Kawamoto H Koizumi H Uchikoshi T . Expression of the G2-m checkpoint regulators cyclin B1 and cdc2 in nonmalignant and malignant human breast lesions: immunocytochemical and quantitative image analyses. Am J Pathol (1997) 150(1):15–23. 9006317 PMC1858517

[24] Soria JC Jang SJ Khuri FR Hassan K Liu D Hong WK . Overexpression of cyclin B1 in early-stage non-small cell lung cancer and its clinical implication. Cancer Res (2000) 60(15):4000–4. 10945597

[25] Hassan KA Ang KK El-Naggar AK Story MD Lee JI Liu D . Cyclin B1 overexpression and resistance to radiotherapy in head and neck squamous cell carcinoma. Cancer Res (2002) 62(22):6414–7. 12438226

[26] Kallakury BV Sheehan CE Rhee SJ Fisher HA Kaufman RP Jr. Rifkin MD . The prognostic significance of proliferation-associated nucleolar protein p120 expression in prostate adenocarcinoma: a comparison with cyclins a and B1, ki-67, proliferating cell nuclear antigen, and p34cdc2. Cancer (1999) 85(7):1569–76. doi: 10.1002/(SICI)1097-0142(19990401)85:7<1569::AID-CNCR19>3.0.CO;2-M 10193948

[27] LaTulippe E Satagopan J Smith A Scher H Scardino P Reuter V . Comprehensive gene expression analysis of prostate cancer reveals distinct transcriptional programs associated with metastatic disease. Cancer Res (2002) 62(15):4499–506. 12154061

[28] Glinsky GV Berezovska O Glinskii AB . Microarray analysis identifies a death-from-cancer signature predicting therapy failure in patients with multiple types of cancer. J Clin Invest (2005) 115(6):1503–21. doi: 10.1172/JCI23412 PMC1136989 15931389

[29] Gomez LA de Las Pozas A Reiner T Burnstein K Perez-Stable C . Increased expression of cyclin B1 sensitizes prostate cancer cells to apoptosis induced by chemotherapy. Mol Cancer Ther (2007) 6(5):1534–43. doi: 10.1158/1535-7163.MCT-06-0727 17513602

[30] Du R Huang C Liu K Li X Dong Z . Targeting AURKA in cancer: molecular mechanisms and opportunities for cancer therapy. Mol Cancer (2021) 20(1):15. doi: 10.1186/s12943-020-01305-3 33451333 PMC7809767

[31] Beltran H Rickman DS Park K Chae SS Sboner A MacDonald TY . Molecular characterization of neuroendocrine prostate cancer and identification of new drug targets. Cancer Discovery (2011) 1(6):487–95. doi: 10.1158/2159-8290.CD-11-0130 PMC3290518 22389870

[32] Otto T Horn S Brockmann M Eilers U Schuttrumpf L Popov N . Stabilization of n-myc is a critical function of aurora a in human neuroblastoma. Cancer Cell (2009) 15(1):67–78. doi: 10.1016/j.ccr.2008.12.005 19111882

[33] Mosquera JM Beltran H Park K MacDonald TY Robinson BD Tagawa ST . Concurrent AURKA and MYCN gene amplifications are harbingers of lethal treatment-related neuroendocrine prostate cancer. Neoplasia (2013) 15(1):1–10. doi: 10.1593/neo.121550 23358695 PMC3556934

[34] Beltran H Oromendia C Danila DC Montgomery B Hoimes C Szmulewitz RZ . A phase II trial of the aurora kinase a inhibitor alisertib for patients with castration-resistant and neuroendocrine prostate cancer: Efficacy and biomarkers. Clin Cancer Res (2019) 25(1):43–51. doi: 10.1158/1078-0432.CCR-18-1912 30232224 PMC6320304

[35] Graff JN Higano CS Hahn NM Taylor MH Zhang B Zhou X . Open-label, multicenter, phase 1 study of alisertib (MLN8237), an aurora a kinase inhibitor, with docetaxel in patients with solid tumors. Cancer (2016) 122(16):2524–33. doi: 10.1002/cncr.30073 27192055

[36] Miralaei N Majd A Ghaedi K Peymani M Safaei M . Integrated pan-cancer of AURKA expression and drug sensitivity analysis reveals increased expression of AURKA is responsible for drug resistance. Cancer Med (2021) 10(18):6428–41. doi: 10.1002/cam4.4161 PMC8446408 34337875

[37] Namekawa T Ikeda K Horie-Inoue K Inoue S . Application of prostate cancer models for preclinical study: Advantages and limitations of cell lines, patient-derived xenografts, and three-dimensional culture of patient-derived cells. Cells (2019) 8(1). doi: 10.3390/cells8010074 PMC6357050 30669516

