# Supplementary material for: CCNB1 and AURKA are critical genes for prostate cancer progression and castration-resistant prostate cancer resistant to vinblastine
Source: Front Endocrinol (Lausanne). 2022 Dec 19;13:1106175. doi: 10.3389/fendo.2022.1106175 (PMC9806262; doi:10.3389/fendo.2022.1106175)
Supplement: Supplementary file 1 [file DataSheet_1.pdf]

Supplementary Material

***CCNB1* and *AURKA* are critical genes for prostate cancer progression and castration-resistant prostate cancer resistant to vinblastine**

Xi Chen[1†], Junjie Ma[2†], Xin'an Wang[1†], Tong Zi[1], Duocheng Qian[3*], Chao Li[1*], Chengdang Xu[1*]

*** Correspondence:**

Duocheng Qain

Email: qiandc666@126.com,

Chao Li

Email: chaoli1979@126.com,

Chengdang Xu

Email: xuchengdang1990@163.com

Supplementary Figures

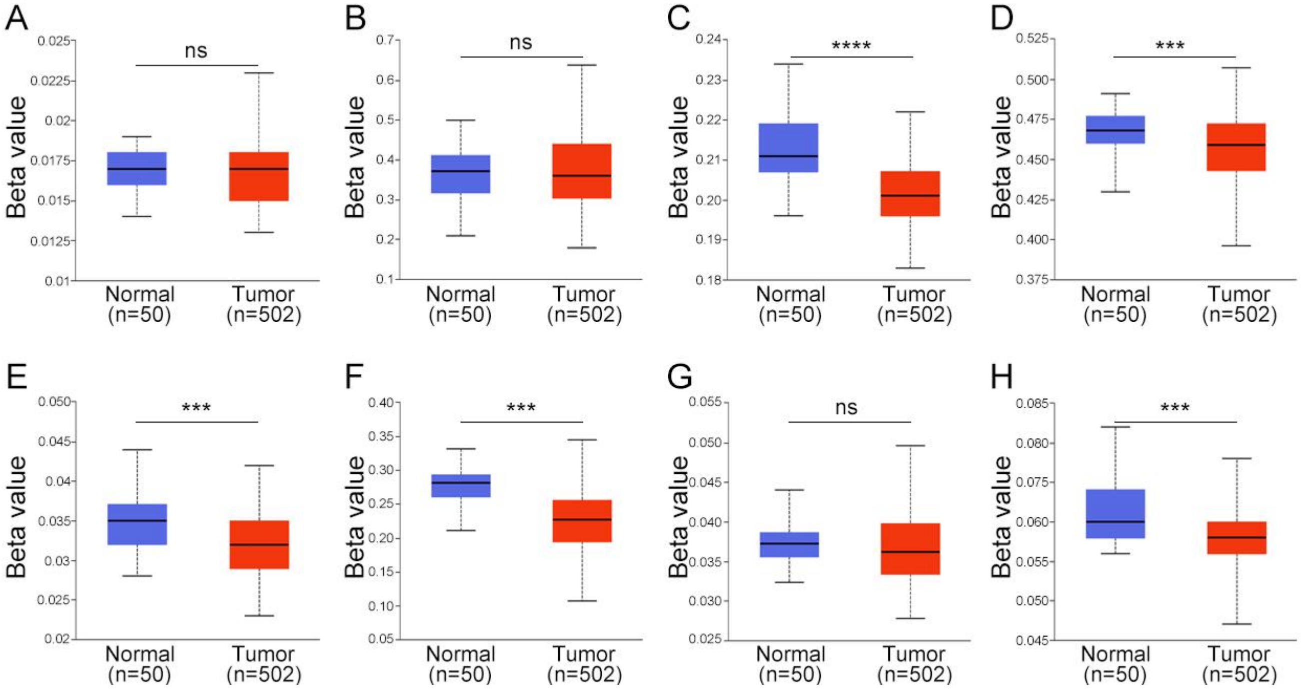

Supplementary Figure 1. The methylation level of vinblastine resistance-related genes in PCa from TCGA database. (A) *CDC20* (B) *CCNB1* (C) *GPSM2* (D) *AURKA* (E) *EBLN2* (F) *CCDC150* (G) *CENPA* (H) *TROAP*. ns represents no statistical differences, *** represents *P*<0.001, **** represents *P*<0.0001.

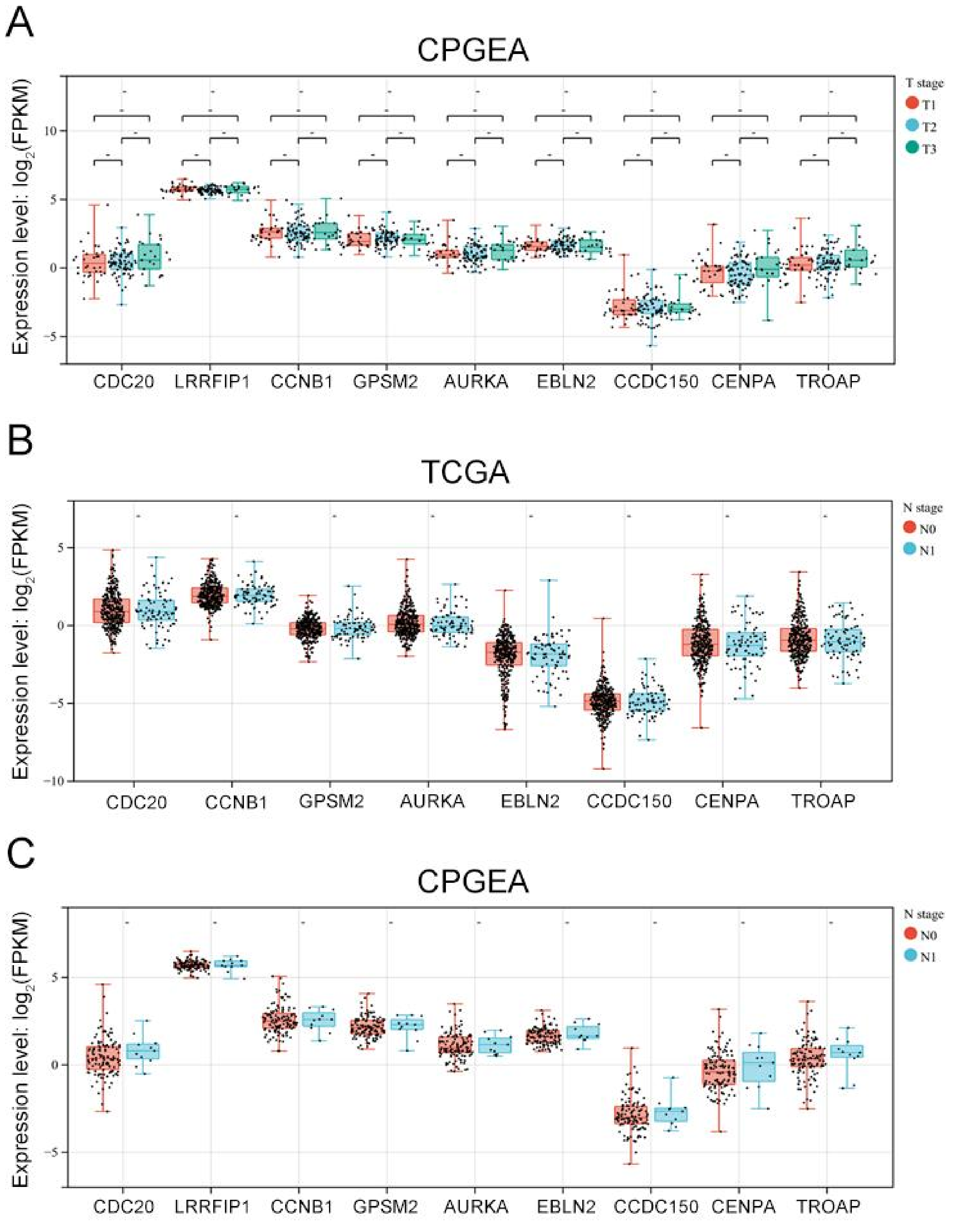

Supplementary Figure 2. The expression of vinblastine resistance-related genes in different tumor stage from different databases. (A) The expression of nine vinblastine resistance-related hub genes in different T stage from CPGEA database. (B) The expression of eight vinblastine resistance-related hub genes in different N stage from TCGA database. (C) he expression of eight vinblastine

resistance-related hub genes in different N stage from TCGA database. – represents no statistical differences.

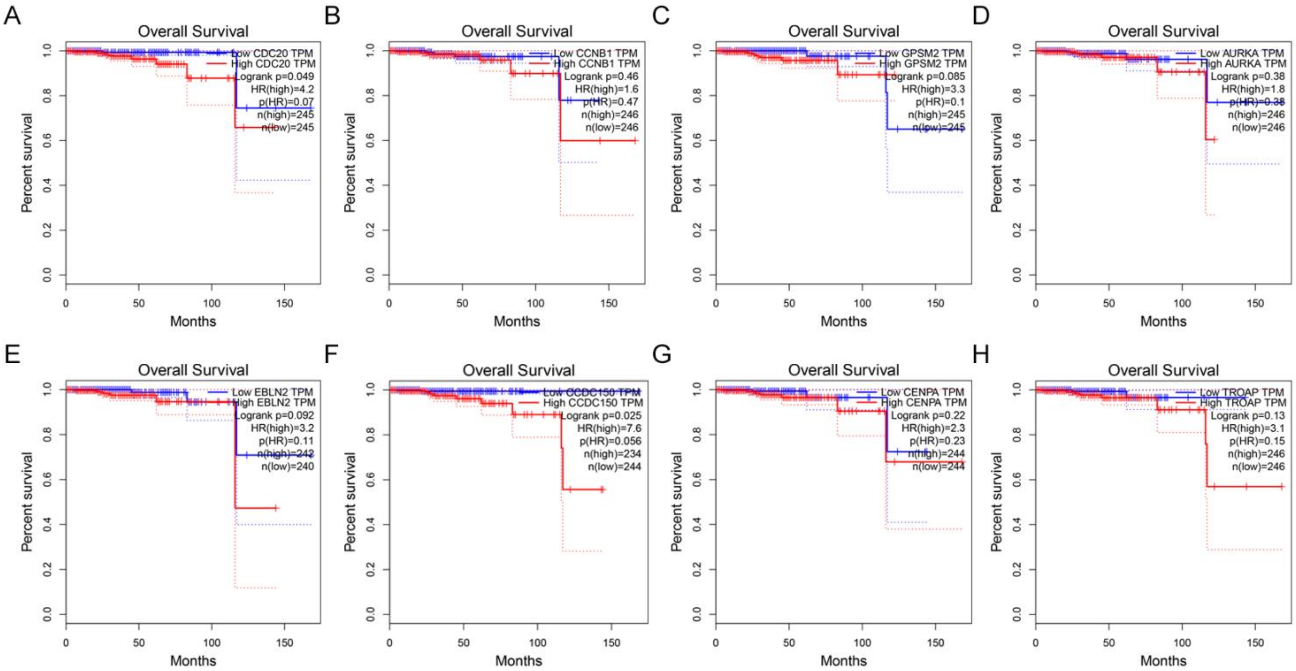

Supplementary Figure 3. The expression of vinblastine resistance-related genes with PCa patients OS status from TCGA database. (A) *CDC20* (B) *CCNB1* (C) *GPSM2* (D) *AURKA* (E) *EBLN2* (F) *CCDC150* (G) *CENPA* (H) *TROAP*

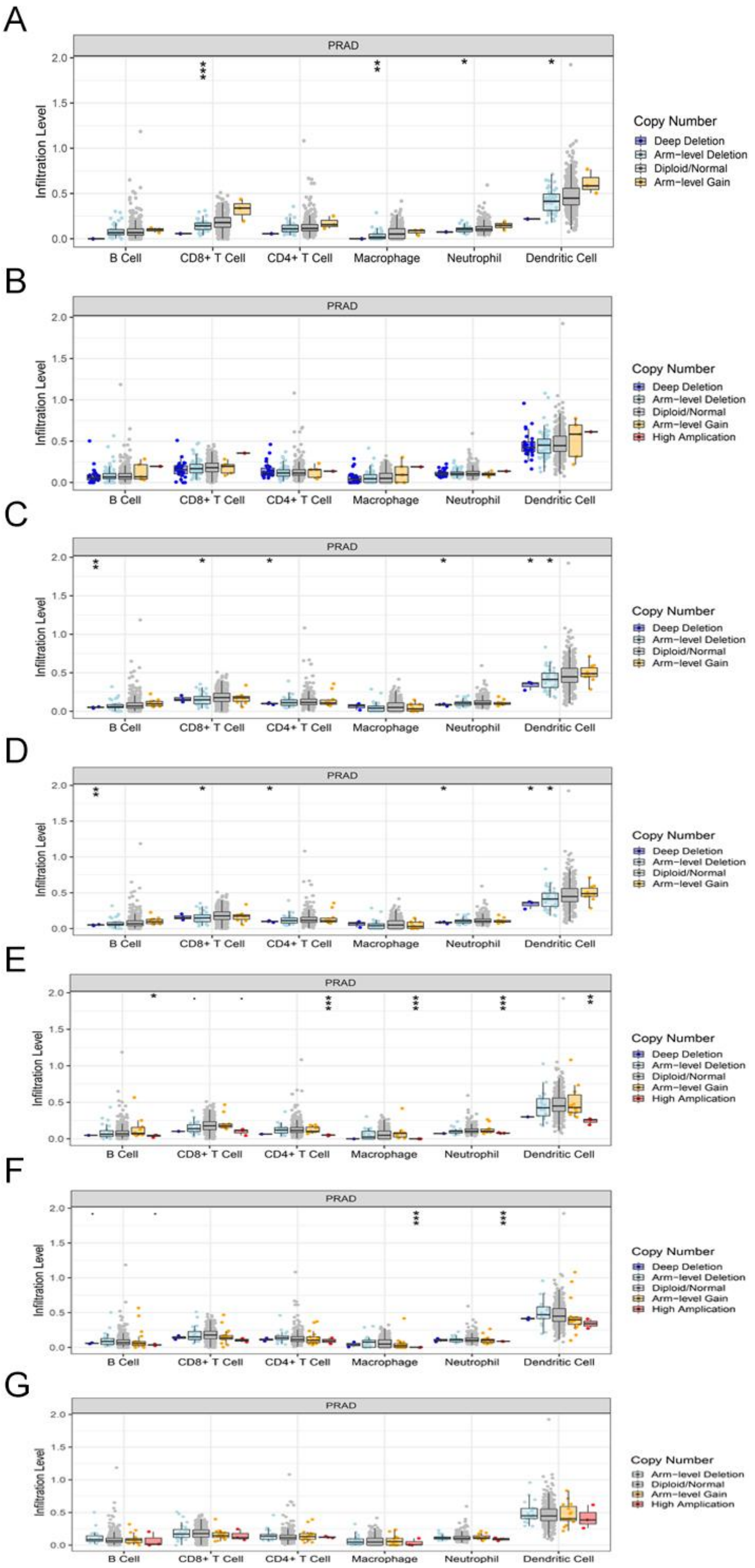
A
PRAD
Infiltration Level
2.0
1.5
1.0
0.5
0.0
B Cell
CD8+ T Cell
CD4+ T Cell
Macrophage
Neutrophil
Dendritic Cell
Copy Number
Deep Deletion
Arm−level Deletion
Diploid/Normal
Arm−level Gain
B
PRAD
Infiltration Level
B Cell
CD8+ T Cell
CD4+ T Cell
Macrophage
Neutrophil
Dendritic Cell
Copy Number
Deep Deletion
Arm−level Deletion
Diploid/Normal
Arm−level Gain
High Amplication
C
PRAD
Infiltration Level
B Cell
CD8+ T Cell
CD4+ T Cell
Macrophage
Neutrophil
Dendritic Cell
Copy Number
Deep Deletion
Arm−level Deletion
Diploid/Normal
Arm−level Gain
D
PRAD
Infiltration Level
B Cell
CD8+ T Cell
CD4+ T Cell
Macrophage
Neutrophil
Dendritic Cell
Copy Number
Deep Deletion
Arm−level Deletion
Diploid/Normal
Arm−level Gain
E
PRAD
Infiltration Level
B Cell
CD8+ T Cell
CD4+ T Cell
Macrophage
Neutrophil
Dendritic Cell
Copy Number
Deep Deletion
Arm−level Deletion
Diploid/Normal
Arm−level Gain
High Amplication
F
PRAD
Infiltration Level
B Cell
CD8+ T Cell
CD4+ T Cell
Macrophage
Neutrophil
Dendritic Cell
Copy Number
Deep Deletion
Arm−level Deletion
Diploid/Normal
Arm−level Gain
High Amplication
G
PRAD
Infiltration Level
B Cell
CD8+ T Cell
CD4+ T Cell
Macrophage
Neutrophil
Dendritic Cell
Copy Number
Arm−level Deletion
Diploid/Normal
Arm−level Gain
High Amplication

Supplementary Figure 4. The correlation between the mutation type of vinblastine resistance-related genes and immune cell infiltration from TIMER webtool. (A) *CDC20* (B) *LRRFIP1* (C) *CCNB1* (D) *GPSM2* (E) *AURKA* (F) *CCDC150* (G) *CENPA* (H) *TROAP* – represents no statistical differences. * represents $P<0.05$, ** represents $P<0.01$, *** represents $P<0.001$.

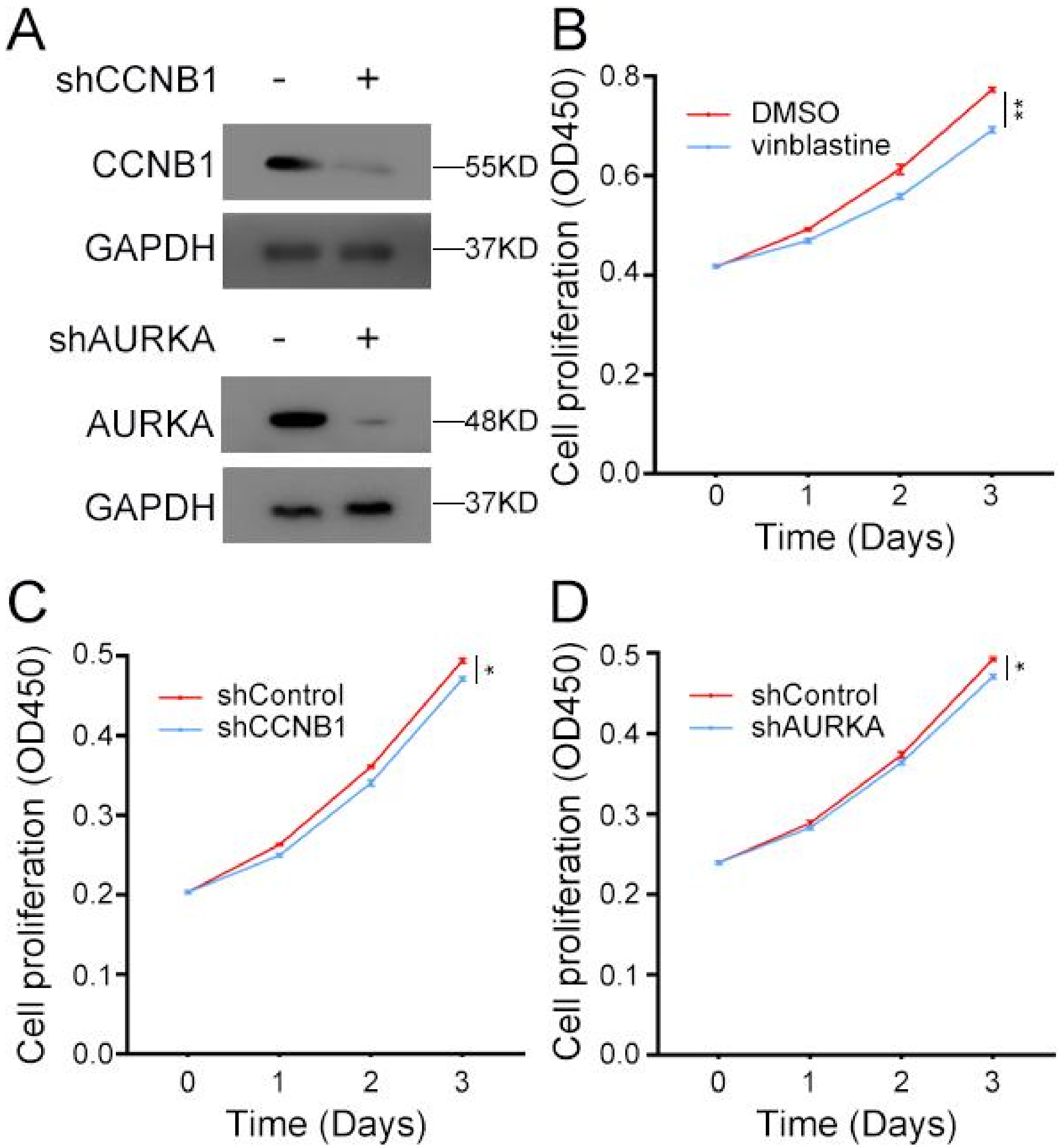

Supplementary Figure 5. The role of *CCNB1* and *AURKA* in influencing 22Rv1 cell proliferation in vinblastine. (A) The protein level of CCNB1 and AURKA when different lentivirus transfected into 22Rv1 CRPC cells. (B) The cell proliferation level of 22Rv1 cells when 22Rv1 cells treated by DMSO or vinblastine. (G-H) The cell proliferation level of 22Rv1 cells after different lentivirus

transfected into 22Rv1 CRPC cells with vinblastine treatment (G) shCCNB1 (H) shAURKA. * represents *P*<0.05, ** represents *P*<0.01.
